# Evaluation of the osteoclastogenic process associated with RANK / RANK-L / OPG in odontogenic myxomas

**DOI:** 10.4317/medoral.22372

**Published:** 2018-04-24

**Authors:** María del Carmen González-Galván, Adalberto Mosqueda-Taylor, Ronell Bologna-Molina, Amaia Setien-Olarra, Xabier Marichalar-Mendia, José-Manuel Aguirre-Urizar

**Affiliations:** 1 Faculty of Dentistry, National University of Asunción, Paraguay. Scholarship by Itaipú Binacional- Paraguay; 2 Health Care Department, Universidad Autónoma Metropolitana Xochimilco, Ciudad de México, México; 3 Faculty of Dentistry, University of the Republic (UDELAR), Uruguay; 4 UFI 11/25. Department of Stomatology II. Faculty of Medicine and Nursing. University of the Basque Country (UPV/EHU), Leioa, Spain

## Abstract

**Background:**

Odontogenic myxoma (OM) is a benign intraosseous neoplasm that exhibits local aggressiveness and high recurrence rates. Osteoclastogenesis is an important phenomenon in the tumor growth of maxillary neoplasms. RANK (Receptor Activator of Nuclear Factor κappa B) is the signaling receptor of RANK-L (Receptor activator of nuclear factor kappa-Β ligand) that activates the osteoclasts. OPG (osteoprotegerin) is a decoy receptor for RANK-L that inhibits pro-osteoclastogenesis. The RANK / RANKL / OPG system participates in the regulation of osteolytic activity under normal conditions, and its alteration has been associated with greater bone destruction, and also with tumor growth.

**Objectives:**

To analyze the immunohistochemical expression of OPG, RANK and RANK-L proteins in odontogenic myxomas (OMs) and their relationship with the tumor size.

**Material and Methods:**

Eighteen OMs, 4 small (<3 cm) and 14 large (> 3cm) and 18 dental follicles (DF) that were included as control were studied by means of standard immunohistochemical procedure with RANK, RANKL and OPG antibodies. For the evaluation, 5 fields (40x) of representative areas of OM and DF were selected where the expression of each antibody was determined. Descriptive and comparative statistical analyses were performed with the obtained data.

**Results:**

There are significant differences in the expression of RANK in OM samples as compared to DF (*p* = 0.022) and among the OMSs and OMLs (*p* = 0.032). Also a strong association is recognized in the expression of RANK-L and OPG in OM samples.

**Conclusions:**

Activation of the RANK / RANK-L / OPG triad seems to be involved in the mechanisms of bone balance and destruction, as well as associated with tumor growth in odontogenic myxomas.

** Key words:**Odontogenic myxoma, dental follicle, RANK, RANK-L, OPG, osteoclastogenesis.

## Introduction

Odontogenic myxoma (OM) is a benign intraosseous neoplasm of the jaws, which exhibits local aggressiveness and high recurrence rates (1). Its frequency ranges between 2.2% and 17.7% of all odontogenic tumors (2-12).

It is characterized microscopically by a monotonous hypocellular proliferation of spindle-shaped or stellate cells embedded in an abundant myxoid extracellular matrix, with little amount of collagen, although some cases may show a greater amount and are called mixofibromas (13-15).

The treatment of choice is conservative or radical surgical excision, depending on the size and location of the tumor (16). Radiological long-term follow-up is mandatory, as recurrence has been reported up to 15 years after surgery (1).

RANK (receptor activator of nuclear factor kappa B) is a member of the family of tumor necrosis factor receptors, and is the signaling receptor of RANK-L (receptor ligand activator for nuclear factor κappa B) (17). The RANK-L homotrimeric protein is typically bound to the membrane of osteoblastic cells and its binding to RANK stimulates the activation of osteoclasts (17,18). Osteoprotegerin (OPG) is a decoy receptor of RANK-L that inhibits pro-osteoclastogenesis through the interaction of RANK-L and RANK, thereby inhibiting bone resorption (17).

The RANK / RANK-L / OPG system participates in the regulation of osteolytic activity under normal conditions, and its alteration is associated with various pathologic conditions, including bone destruction associated to tumor growth (19). The interaction between RANK and RANK-L plays a critical role in the production, differentiation and activation of osteoclasts, which leads to bone resorption (20,21).

The purpose of this study was to analyze and compare the immunohistochemical expression of RANK, RANK-L and OPG proteins in odontogenic myxoma and its relationship with tumor size.

## Material and Methods

Tissue samples from 18 inflammation-free odontogenic myxomas (10 women and 8 men, mean age 32.83 years, range 10 - 53) and 18 inflammation-free dental follicles (9 women and 9 men, mean age 14. 4 years, range 9 - 22) diagnosed at the Oral Pathology Laboratory of the Universidad Autonoma Metropolitana Xochimilco and a private Oral Pathology Service in Mexico City were included in the present study.

This study was approved by the Division of Biological and Health Sciences of the Universidad Autonoma Metropolitana Xochimilco (Mexico).

The size of the OMs included in this study was obtained from radiographic interpretation stated in the clinical files. OM samples were classified as small myxomas (OMS) when these were up to 3 cm (N = 4), and large myxomas (OML) when greater than 3 cm (N = 13) in its larger dimension. No data on the size of the tumor could be obtained from one of the odontogenic myxomas.

The histopathological diagnosis in each case was confirmed in sections stained with H&E and was based on microscopic criteria described in the most recent WHO Histological Classification of Tumors of the Head and Neck (22).

Two microns sections were obtained from each paraffin block, which were subsequently dewaxed, hydrated and treated with 0.1 mol / L sodium citrate (pH 6.2) to expose the antigenic epitopes. The endogenous peroxidases were blocked with 0.9% hydrogen peroxide. Incubation of the primary antibody was carried out in humidity chambers (Sequenza ™ Slide Rack), with a 1: 150 dilution of the OPG polyclonal antibody (Gene Tex®, USA, 45 minutes); 1: 500 of the RANK polyclonal antibody (Gene Tex®, USA, 45 minutes) and 1: 800 of the RANK-L polyclonal antibody (Gene Tex®, USA, 45 minutes). Antibody dilutions were carried out with the diluent S2022 (DAKO®, Carpintería, Ca, USA). The reaction was visualized with the Mouse Rabil Immunodetector system (BioSB ®, Santa Bárbara, Ca, USA), and revealed with 3.3 diaminobenzidine-hydrochloride (DAB) producing a brown precipitate. The contrast of the sections was performed with Gill’s hematoxylin and the assembly with a permanent medium (Eukitt®). Positive and negative controls were made for each antibody.

A qualitative and semiquantitative assessment of the histological and immunohistochemical aspects was performed with an Olympus® microscope (CX31), analyzing 5 fields of 40x in each case, using the Image J® program to count cells. The assessment of the immunohistochemical expression was carried out by 3 oral pathologists in an individualized manner and subsequently a common consensus was reached. The evaluation criteria were based on previous studies (18,19,23).

**Figure 1:**
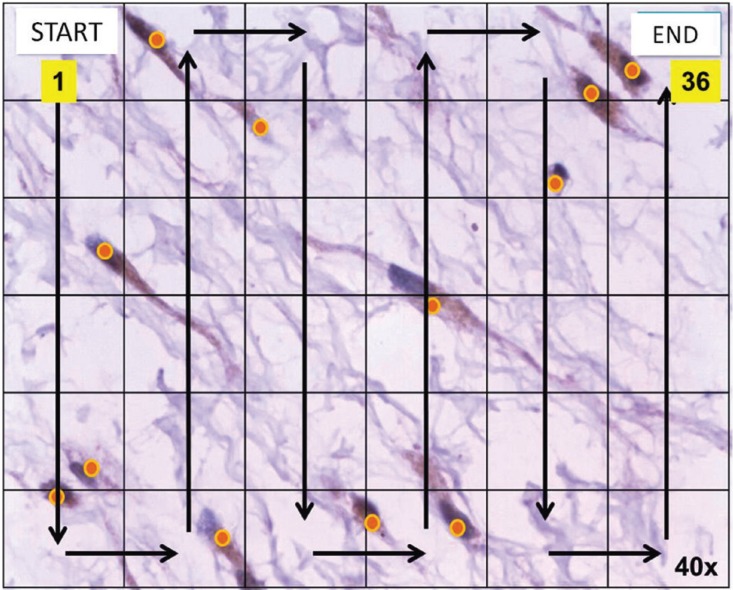
Method employed of the positive cell counting (40×).

The rack proposed by Bologna-Molina *et al.* (24) was used for the analysis of the immunohistochemical expression. Then, with a digital camera (Olympus® CX 31) five microphotographs of the most representative areas were taken at 40x. Subsequently, a 6x6 rack was placed on each photograph. In each image, the start of cell counting was made from the upper left frame and culminated in the upper right margin, following the same order (Fig. 1). In this way, the manual counting of the number of positive and negative cells in each box of the rack was made, helped by the Image J® software.

Statistical analyzes carried out in this study, descriptive and comparative, were performed using SPSS v15.0 (SPSS Inc®, Chicago, ILL, USA). The examiners were standardized and a Kappa index of 0.88 was obtained. The comparison of the means of the immunohistochemical marker was performed with the Mann-Whitney U-test and the Kruskal-Wallis test, and *p*-values <0.05 were considered statistically significant.

## Results

 Table 1 shows the results found in relation to the immunohistochemical markers analyzed. When compared to DF, only RANK displayed a significant expression in OM samples (*p* = 0.022). Specifically, RANK shows lower expression in the OM group. Additionally, the OPG marker is close to the statistically significant difference among OM and FD groups.

**Table 1:**
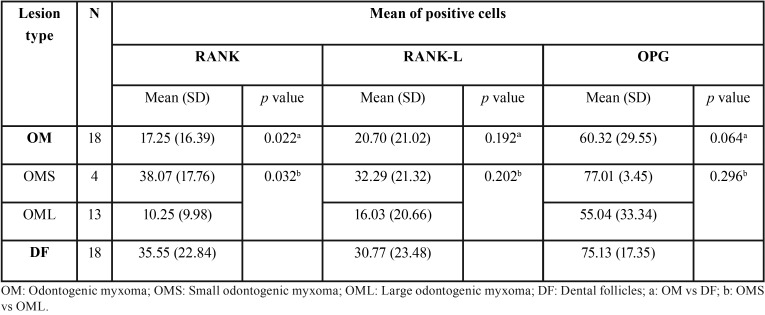
Average expression of RANK, RANK-L and OPG in odontogenic myxomas (OM) and dental follicles (DF).

When analyzing the expression levels of the 3 selected markers, we found statistically significant differences in RANK marker (*p* = 0.032), observing a lower mean expression in the OML group in relation to the OMS group. When comparing the levels of expression of the other two markers, RANK-L and OPG, we did not find statistically significant differences (*p* = 0.202 and *p* = 0.296 respectively), however these two markers have similar patterns of expression as in RANK, i.e., OML samples presented a higher expression.

 Table 2 shows the correlation between the different markers. In odontogenic myxomas, there is a strong correlation among RANK-L and OPG markers (*p* = 0.002) and not so strong among RANK and OPG markers (*p* = 0.021). On the other hand, there was not a statistically significant correlation among RANK and RANK-L markers (*p* = 0.069).

**Table 2:**
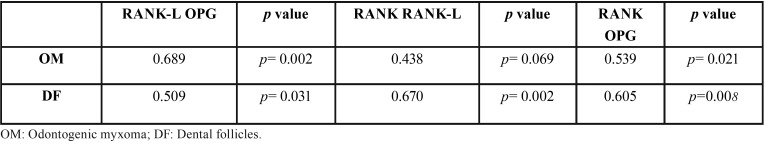
Spearman’s Correlation Test on the Expression of RANK-RANK-L-OPG in odontogeic myxomas (OM) and dental follicles (DF).

Finally, in relation to dental follicles, the 3 ratios, RANK-L / OPG (*p* = 0.031) RANK / RANK-L (*p* = 0.002) and RANK / OPG (*p* = 0.008) displayed statistically significant correlations.

## Discussion

Previous studies (18,19,23,25) have shown that the RANK / RANK-L / OPG system is abnormally regulated in several osteolytic diseases, including neoplastic and non-neoplastic odontogenic lesions, where a higher expression of RANK-L or a decrease in OPG levels seem to play an important role in bone destruction (19,21,25). The process of tumor osteolysis has been associated with changes in the levels of RANK-L and OPG in multiple myeloma, osteosarcoma, osteoblastic metastasis of prostate carcinoma, giant cell lesions, and interestingly, in some odontogenic tumors, such as ameloblastoma, odontogenic keratocyst and calcifying epithelial odontogenic tumor (18,23,25).

In 2008 Andrade *et al.* carried out the first study that suggests that the alteration in the RANK / RANK-L / OPG signaling pathway could be related to the bone loss that occurs in various odontogenic tumors (23). Among the lesions studied, 7 OMs were included, 4 of which exhibited a greater expression of RANK-L in relation to OPG. Our results were contrary to this, observing a greater positivity in OPG (94.4%) in relation to RANK-L (66.6%). There are some points that need to be taken into consideration in order to explain these differences; first, these dissimilarities could be related to the greater number of cases in our study and their analysis according to different sizes, as well as differences in the methodology employed, which are fundamental elements in these investigations.

On the other hand, although we did not find significant differences in relation to the size of the lesions, it is important to mention that in OMS there was a smaller expression of OPG and RANK-L. These data could suggest that while increasing the size of OMs, osteoclastogenic activity tend to increase, which could be related to clinical aspects, since OM is a neoplasm that grows more slowly through bone tissue as compared to other locally infiltrating tumors. In addition, it could be suggested that the differences observed in the size of the sample, the stage (size) of the lesions at the time of the diagnosis and the presence of inflammation present in previous studies (19,23) could also help to explain the disparities observed in relation to our findings.

Moreover, we observed in our study that DF samples have a higher average of positive cells for RANK, RANK-L and OPG than OM samples, but RANK was the only marker that showed statistically significant differences (*p*<0.05), independently of the size of the OMs. In addition, we have found a strong association between RANK-L and OPG in OM samples, and among RANK / RANK-L in DF samples. These findings suggest that the modification produced in this pathway would be one of the mechanisms by which this benign but aggressive odontogenic neoplasm can grow and invade the adjacent tissue as it reaches a larger size.

In this regard, our findings and those of Andrade *et al.* (23) indicate that most of the odontogenic tumors present variations among RANK-L and OPG expression, possibly dependent on the type of tumor, mechanisms of growth, and size, which has been interpreted as active and inactive stages of tumor growth and stages of interference with tumor osteolysis (19).

In order to compare among those studies that have evaluated these markers, it is necessary to have a standardized method that is objective and reproducible in all cases, since the differences found in some studies (23,26,27) could be due to different evaluation criteria, sampling, technical conditions or tissue preservation. For this reason, in our study we have used the method proposed by Bologna-Molina *et al.* (24), through which the cell counting could be performed in an objective manner. The standardization of this method could be of great relevance for studies aimed to find new therapeutic strategies to reduce the size or growth of this type of lesion.

Based on the fact that Qian *et al.* (26) found that OPG suppresses both, osteoclastogenesis induced by ameloblastoma and bone resorption caused by osteoclasts, De Matos *et al.* (27) have suggested that OPG could be used as therapeutic treatment of ameloblastoma in the future to stop bone loss and thus, minimize the extent of bone destruction before surgical management (23).

Different studies performed by our group (14,28,29) have shown that the growth of the odontogenic myxoma is the product of a complex multifactorial process in which cell proliferation and angiogenesis are involved mainly in the early stages. With the present results, we suggest that the growth of these neoplasms can also be related with the production and performance of different mechanisms related with the RANK / RANK-L / OPG system. The increase of the tumor size would modify the production of these proteins, modifying their growth capacity.

In summary, our results suggest that the RANK / RANK-L / OPG triad may be involved in the mechanisms of bone balance and destruction of odontogenic myxomas. These data also suggest that the modification produced in this signaling pathway would be one of the main mechanisms by which this odontogenic neoplasm grows and invades the adjacent tissues.
